# To drain or not to drain: the association between residual intraperitoneal gas and post-laparoscopic shoulder pain for laparoscopic cholecystectomy

**DOI:** 10.1038/s41598-021-85714-4

**Published:** 2021-06-01

**Authors:** Shun-Chin Yang, Kuang-Yi Chang, Ling-Fang Wei, Yi-Ming Shyr, Chiu-Ming Ho

**Affiliations:** 1 Department of Anesthesiology, Taipei Veterans General Hospital and National Yang-Ming University, Taipei, Taiwan; 2 National Central Library, Taipei, Taiwan; 3 Division of General Surgery, Department of Surgery, Taipei Veterans General Hospital and National Yang-Ming University, Taipei, Taiwan; 4 Department of Anesthesiology, Taipei Veterans General Hospital, No. 201, Sec. 2, Shipai Rd., Beitou District, Taipei, 11217 Taiwan

**Keywords:** Health care, Medical research, Risk factors

## Abstract

Residual intra-peritoneal gas may be associated with post-laparoscopic shoulder pain (PLSP), which is a frequently and disturbance compliant after surgery. Herein, we aimed to examine whether expiring residual gas via a surgical drain reduces the frequency and intensity of PLSP in the first day after laparoscopic cholecystectomy. 448 participants were enrolled in this prospective cohort study. The incidence and severity of PLSP after surgery were recorded. Of these, the cumulative incidence of PLSP in the drain group was lower particularly at the 12th postoperative hour (18.3% vs. 27.6%; *P* = 0.022), 24th postoperative hour (28.8% vs. 38.1%; *P* = 0.039), and throughout the first postoperative day (*P* = 0.035). The drain group had less severe PLSP (crude Odds ratio, 0.66; *P* = .036). After adjustment using inverse probability of treatment weighting, the drain group also had a significant lower PLSP incidence (adjusted hazard ratio = 0.61, *P* < 0.001), and less severe PLSP (adjusted odds ratio = 0.56, *P* < 0.001). In conclusion, the maneuver about passive force to expel residual gas, surgical drain use, contributes to reduce the incidence and severity of PLSP, suggesting that to minimize residual gas at the end of surgery is useful to attenuate PLSP.

## Introduction

Laparoscopic cholecystectomy (LC) is the most popular laparoscopic surgery worldwide, and has evolved to become the standard treatment for symptomatic calculous and acalculous cholecystitis^1^. LC has several advantages, including a smaller wound, fewer post-operative respiratory complications, and shorter convalescence and duration of hospital stay^2^. Post-laparoscopic shoulder pain (PLSP), however, is an easily neglected but not rare complaint following LC, with a reported incidence of up to approximately 50%^3^. This symptom not only leads to greater discomfort and anxiety during recovery periods, but also prolongs hospitalization and can even result in readmission^4^. Thus, finding methods to reduce PLSP after LC is central to be explored.

Surgical drains are generally used in abdominal surgeries for therapeutic purposes, including to monitor the drainage of abdominal effusion in order to avoid bleeding or the formation of intra-abdominal abscesses, and to remove debris; however, the use of surgical drain is related to increasing wound infection rates and more post-operative abdominal pain^5,6^. Notably, residual intra-peritoneal gas is also evacuated through a drain. Evidence suggests that the mechanism of PLSP concerns the irritation of the phrenic nerve by residual gas inflation during laparoscopic surgery, although the complete mechanism of PLSP remains unclear^7,8^. Remarkably, several studies have shown that patients with a drain have less PLSP after a laparoscopic procedure, suggesting that carbon dioxide (CO_2_) flowing out via the site of the drain might decrease the irritant effects of residual gas in the peritoneal cavity^9–11^. However, other studies have reported different results showing that a drain does not affect PLSP incidence after LC^12,13^. The role of drains in reducing PLSP after LC, therefore, still remains controversial.

In larger either prospective or retrospective observational studies, differences in patient characteristics will potentially confound the results. Increasing evidence in many fields of research demonstrates that such differences can be adjusted using propensity score analysis^14^. Our hypothesis was that the maneuver about passive expel residual intra-peritoneal gas, the placement of surgical drain, reduced PLSP in the early postoperative periods. The objective of this prospective cohort study was to examine whether the use of a drain reduces the incidence or severity of PLSP in the first 24 h after LC, while using propensity scores to account for selection bias.

## Methods

### Study design and population

This prospective cohort study was conducted in a single medical center and included men and non-pregnant women admitted for scheduled LC between March, 2010 and February, 2011. It followed the Strengthening the Reporting of Observational Studies in Epidemiology guidelines^15^. Patients who had major complications, including biliary tract injury and massive bleeding, were excluded, because postoperative evaluation of these patients was difficult. Patients with a history of shoulder pain, upper laparotomy, other abdominal surgical procedures, or conversion to laparotomy were also excluded.

### Anesthesia and surgery

Standard vital signs were measured, including by electrocardiogram, non-invasive blood pressure monitoring, and pulse oximetry, before any drugs were administered. Intravenous sodium thiopental, propofol, or etomidate were used for anesthetic induction in all patients, with the drug chosen according to clinical judgment. Muscle relaxation was induced using intravenous rocuronium or cis-atracurium to facilitate intubation and maintain anesthesia. The trachea was intubated via an oral route once a sufficient depth of anesthesia was reached. Intraoperative analgesia was achieved by intravenous administration of fentanyl (2–5 μg kg^−1^). Anesthesia was maintained with inhaled volatile anesthetics including isoflurane, desflurane, or sevoflurane to keep end-tidal concentration around one minimum alveolar concentration in a mixture of oxygen and air. The patients’ lungs were mechanically ventilated to maintain normocapnia (end-tidal CO_2_: 4–5.3 kPa). After surgery was completed, pain was controlled with systemic non-steroidal analgesics and opioids. Post-operative pain was managed with intravenous meperidine 40 mg once in the post-anesthesia care unit and oral acetaminophen 500 mg every 8 h in the ward, as an analgesic for patients with a verbal rating scale ≥ 4.

The standard four-port LC procedure, as in our previous studies, was performed via four ports in all patients^16,17^. One 12 mm port was implanted through the umbilicus, and a pneumoperitoneum was created by CO_2_ inflation through a Veress needle^18^. Another 12 mm port was inserted below the xiphoid process, and two 5-mm ports were inserted in the subcostal region. During the process of pneumoperitoneum creation, intra-abdominal pressure, volume of gas, and gas flow rate were monitored. Gas pressure was set below 2 kPa, and gas flow did not exceed 2 L min^−1^. The decision to use a J-VAC drain tube (Ethicon, Inc., NJ, USA) was made by the surgeon. The drain was removed 24–48 h after surgery in the absence of bile and blood.

### Data collection

We collected demographic data and took preoperative records of medical history, age, sex, weight, height, and American Society of Anesthesiologists (ASA) physical status classification. Intra-operative records included intra-operative opioid dosage, duration of surgery, duration of CO_2_ inflation, and usage of a drain. Post-operative data included PLSP intensity and post-operative analgesic doses. The primary outcome of this research was PLSP, which was defined as the sensation of pain in the shoulder within 24 h of LC. All enrolled patients were interviewed by the anesthetist the following morning or afternoon (at least 24 h after surgery). Pain was assessed by asking patients to rate their present intensity of PLSP at rest. The precise location and onset time of PLSP were recorded. The severity of pain was assessed using a numeric verbal rating scale (VRS) ranging from 0 (no pain) to 10 (worst pain possible). In this study, we considered a VRS score between 0 and 3 to indicate mild pain, between 4 and 6 moderate pain, and between 7 and 10 severe pain^19^. All of these variables were used for the analyses.

### Sample size

According to the findings of Sandhu and colleagues^20^, the incidence of PLSP was about 56%. Based on clinical observation in our institution and the previous research^21^, we hypothesized that surgical drain insertion after LC reduced the incidence of PLSP by 25%, and the ratio of patients in the surgical drain insertion group to the non-drain group after LC was about 2:3. Assuming a significance level of 0.05, power of 0.8, and a two-sided test, we determined that at least 415 patients should be recruited in this study (NQuery Advisor 4.0; Statistical Solutions Ltd., Cork, Ireland).

### Statistics analysis

On the basis of surgical drain used, the patients were classified into one of two groups. Continuous variables were expressed as mean with standard deviation, and categorical variables were presented as count with percentage. Standardized differences were used to assess balance in observed covariates between the two groups before and after inverse probability of treatment weighting (IPTW) adjustment. Severity and cumulative incidence of PLSP were compared between the two groups using a Mann–Whitney U test and a log rank test, respectively. Since the data collected in this study were not random, and patient characteristics may differ between the two groups, IPTW, a propensity score-based method, was used to compensate for any potential imbalance in the measured variables, as well as to eliminate any possible confounding effects^14^. In our study, propensity scores were generated using a logistic regression model (Supplementary Table S1). In this model, the use of a surgical drain was regarded as the dependent variable and the other measured variables were considered the predictors. The inverse of the estimated probability of surgical drain use was then applied to the following weighted Cox regression analysis. In order to minimize the impact of large weights on analytical results, one percent of cases at the end of the weighting distribution were truncated. The weighted Cox regression analysis was conducted to evaluate the association between surgical drain use and PLSP. Hazards ratios (HRs) with 95% confidence intervals (CIs) were calculated to estimate the association between surgical drain use and PLSP incidence. Furthermore, in order to evaluate the association between surgical drain use and the severity of PLSP, ordinal logistic regression analyses were conducted with IPTW adjustment. The association between surgical drain use and PLSP severity was estimated by the calculation of odds ratios (ORs) with 95% CIs. Sensitivity analyses were performed with multivariable Cox and ordinal logistic regression models, which used surgical drain use and all the other measured variables as predictors. The proportional hazards and odds assumptions were assessed using log–log survival plots and the score test, respectively^22,23^. A value of *P* < 0.05 was considered statistically significant. All the statistical analyses were performed using SAS software, version 9.4 (SAS Institute Inc., Cary, NC, USA).

### Ethical considerations

This study was approved by the local medical ethics review committee (Institutional Review Board, Taipei Veterans General Hospital, Taiwan) and was registered in the ClinicalTrials.gov database (No. NCT01095536, first posted date: March 30, 2010) at February 8, 2010. Written informed consent was obtained from each enrolled patient.

## Results

**Figure 1:**
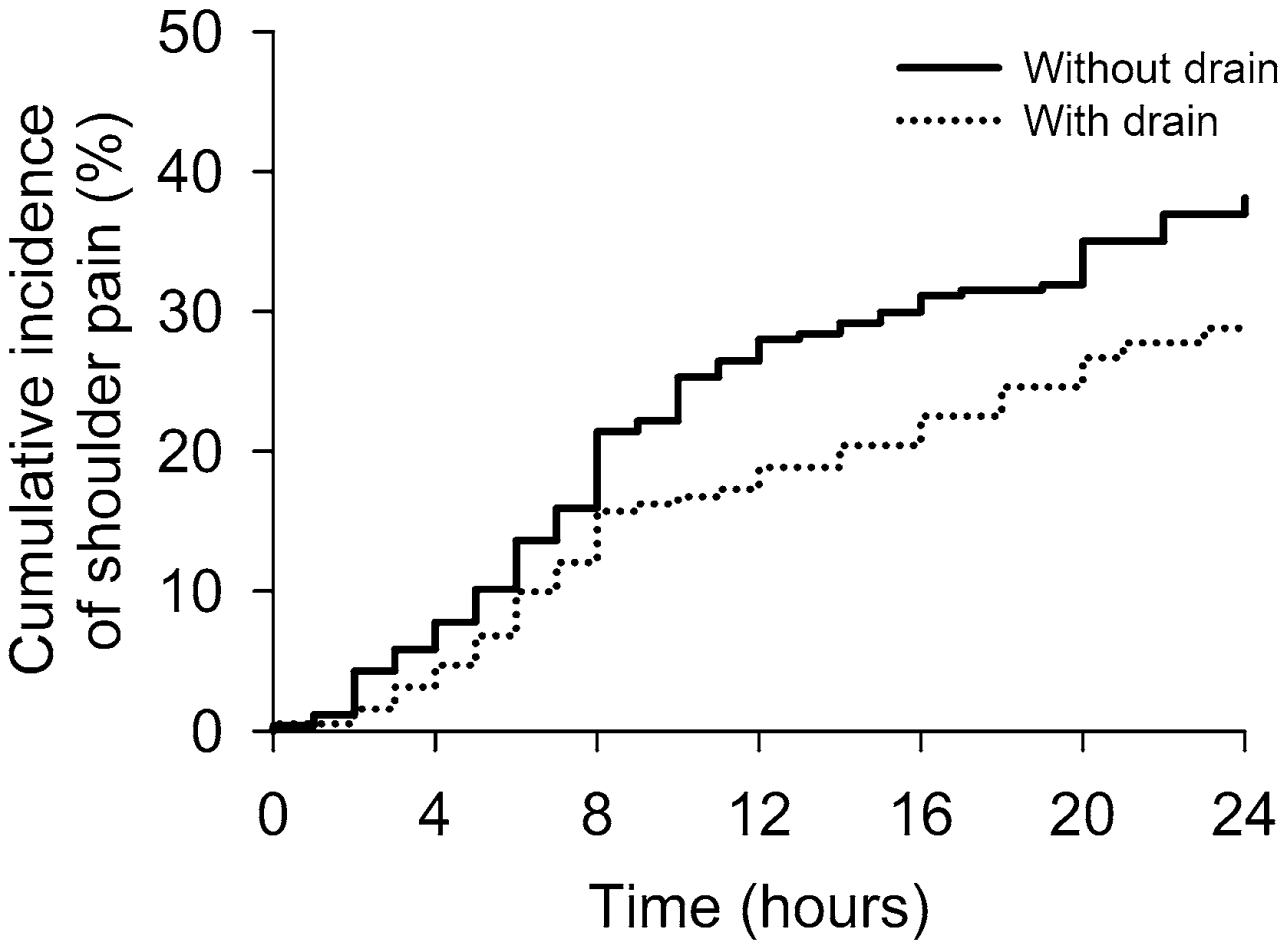
Cumulative incidence of shoulder pain of the groups with drain and without drain.

A total of 469 patients who received LC were enrolled in this study. Of these, 21 patients were excluded, as they converted to laparotomy. A total of 448 patients (58.0% female; mean age [standard deviation, SD], 56 [16] years) were therefore included in the final analysis. The median length of hospital stay was 4 days. Of these, 191 (42.6%) patients received a drain (50.3% female; mean age [SD], 61 [16] years), while 257 (57.3%) patients did not (63.8% female; mean age [SD], 53 [15] years). Increased age and prolong CO_2_ inflation time were related to the placement of surgical drain (Supplementary Table S1). The median time to remove surgical drain was post-operative day 2. The median hospital stay was 5 days in drain group, and 4 days in non-drain group. A total of 153 (34.1%) patients had PLSP. The unadjusted incidence of PLSP in the group with a drain was significantly lower than in the group without a drain (28.8% vs. 38.1%; *P* = 0.039). The cumulative incidence of PLSP in the drain group was lower than that of the non-drain group throughout the first postoperative day (by log rank test, *P* = 0.035) and was found to be significantly lower particularly at the 12th postoperative hour (18.3% vs. 27.6%; *P* = 0.022) and 24th postoperative hour (28.8% vs. 38.1%; *P* = 0.039). Figure 1 compares the cumulative incidence of PLSP between the two groups. Baseline characteristics of the two groups, before and after IPTW adjustment, are compared in Table 1. Before IPTW, the distribution of age, sex, diabetes, hypertension, operation time, and CO_2_ inflation time were unbalanced between the two groups. However, after IPTW, no imbalance in the distributions of measured variables was noted between the two groups.

**Table 1:** Comparisons of baseline characteristics between the groups with drain and the other without drain before and after IPTW adjustment.

	Before IPTW					After IPTW				
	Without drain (N = 257)		With drain (N = 191)		SDD	Without drain (N = 442)		With drain (N = 438)		SDD
Age (year)	53	(15)	61	(16)	51.7	56	(15)	57	(16)	6.4
Sex (F)	164	63.8%	96	50.3%	27.6	266	60.2%	254	58.0%	4.4
Height (cm)	161	(9)	161	(9)	7.2	161	(9)	161	(9)	0.4
Weight (kg)	65	(12)	66	(12)	14.0	65	(12)	66	(11)	2.9
BMI	24.9	(3.7)	25.3	(3.5)	12.6	25.2	(3.7)	25.3	(3.3)	4.0
ASA physical status ≥ 3	23	8.9%	28	14.7%	17.8	48	10.8%	49	11.2%	1.4
Diabetes	21	8.2%	32	16.8%	26.2	53	11.9%	54	12.4%	1.4
Hypertension	64	24.9%	68	35.6%	23.5	131	29.5%	126	28.9%	1.4
Smoking	30	11.7%	29	15.2%	10.3	55	12.3%	54	12.3%	0.2
Fentanyl dose > 2.5 μg kg^−1^	124	48.2%	93	48.7%	0.9	212	47.9%	212	48.4%	1.0
Operation time > 2 h	97	37.7%	128	67.0%	61.3	222	50.2%	227	51.9%	3.4
CO_2_ inflation time > 1 h	61	23.7%	111	58.1%	74.6	166	37.6%	174	39.7%	4.4

Categorical data are showed as count with percentage and parametric data are presented as mean with (standard deviation).

*IPTW* inverse probability treatment weighting, *SDD* standardized difference expressed in percentage, an absolute value greater than 20 indicates imbalance, *BMI* body mass index.

In the univariate analysis, surgical drain use was associated with a reduced risk of PLSP (crude HR = 0.71; 95% CI, 0.51–0.98; *P* = 0.039; Table 2). The findings were similar to that of the weighted Cox regression analysis after IPTW adjustment, which also demonstrated a significant reduction in PLSP risk with surgical drain use (adjusted HR, 0.61; 95% CI, 0.48–0.77; *P* < 0.001). Regarding the severity of PLSP, surgical drain use was associated with less severe PLSP (crude OR, 0.66; 95% CI, 0.44–0.97; *P* = 0.036; Table 2) in the univariate analysis. Similarly, a significant association between surgical drain use and PLSP severity was also found in the weighted Cox regression analysis (adjusted OR, 0.56; 95% CI, 0.43–0.74; *P* < 0.001), after IPTW adjustment.

**Table 2:** Comparisons of the incidence and severity of PLSP between the groups with drain and the other without drain before and after IPTW adjustment.

PLSP	Before IPTW		*P*	After IPTW		*P*
	Without drain	With drain		Without drain	With drain	
	(N = 257)	(N = 191)		(N = 442)	(N = 438)	
Incidence			0.039			< 0.001
	98 (38.1%)	55 (28.8%)		175 (39.5%)	114 (26.1%)	
	Crude HR = 0.71 (0.51–0.98)			Adjusted HR = 0.61 (0.48–0.77)		
Severity			0.036			< 0.001
Mild	44 (17.1%)	24 (12.6%)		85 (19.2%)	44 (10.2%)	
Moderate	36 (14.0%)	26 (13.6%)		57 (13.0%)	61 (14.0%)	
Severe	18 (7.0%)	5 (2.6%)		32 (7.3%)	9 (1.9%)	
	Crude OR = 0.66 (0.44–0.97)			Adjusted OR = 0.56 (0.43–0.74)		

Data are exhibited as count (%). *IPTW* inverse probability of treatment weighting, *HR* hazard ratio, *OR* odds ratio, HR and OR are estimated using Cox and Ordinal logistic regression models, respectively, and presented in their estimated values (95% CI).

Table 3 shows the results of the multivariable Cox regression analysis used for analyzing sensitivity. The association between surgical drain use and reduced PLSP incidence was found to be significant (adjusted HR, 0.68; 95% CI, 0.47–0.99; *P* = 0.045), especially after the multivariable adjustment that took all of the measured variables into account. Moreover, the association between surgical drain use and less severe PLSP remained significant after the multivariable adjustment as well (adjusted OR, 0.63; 95% CI, 0.40–0.99; *P* = 0.046). In the multivariate analyses, a greater height was also associated with an increased incidence and severity of PLSP. Prolonged the operation time (> 2 h), as a sign of difficult LC, was not associated with the incidence and severity of PLSP. Additionally, a longer CO_2_ inflation time was another predictor of a higher PLSP risk (adjusted HR, 1.57; 95% CI, 1.01–2.43; *P* = 0.043), and a lower body weight and ASA physical status > 3 were associated with a higher severity of pain in PLSP.

**Table 3:** Multivariable analysis of miscellaneous factors on the incidence and the more pain severity of PLSP.

	Incidence			Severity		
	HR	95% CI	*P*	OR	95% CI	*P*
Drain	0.68	0.47–0.99	0.045	0.63	0.40–0.99	0.046
Age	0.99	0.98–1.00	0.136	0.99	0.97–1.01	0.209
Sex (M vs. F)	0.93	0.59–1.47	0.757	0.78	0.43–1.39	0.392
Height	1.04	1.01–1.07	0.015	1.06	1.02–1.10	0.002
Weight	0.98	0.97–1.00	0.083	0.97	0.95–1.00	0.019
ASA (≥ 3 vs. < 3)	1.71	1.00–2.94	0.050	2.06	1.05–4.03	0.036
Diabetes	0.91	0.51–1.62	0.752	0.98	0.50–1.93	0.951
Hypertension	1.10	0.73–1.66	0.648	1.09	0.66–1.81	0.726
Smoking	1.06	0.67–1.69	0.794	1.06	0.58–1.91	0.858
Fentanyl dose (> 2.5 vs. ≤ 2.5 μg kg^-1^)	1.04	0.73–1.48	0.821	1.08	0.69–1.68	0.730
Operation time (> 2 vs. ≤ 2 h)	0.83	0.55–1.27	0.395	0.83	0.50–1.38	0.478
CO_2_ inflation time (> 1 vs. ≤ 1 h)	1.57	1.01–2.43	0.043	1.62	0.95–2.76	0.075

*HR* hazard ratio, *OR* Odds ratio.

## Discussion

Surgical drain use significantly reduced the frequency (adjusted HR = 0.61) and intensity (adjusted OR = 0.56) of PLSP in the first 24 h after LC in this prospective cohort study of 448 patients. Moreover, the adjusted results, which were analyzed with IPTW based on propensity scores, were similar to the unadjusted results, suggesting that the selection bias generated in cohort studies did not affect the final results in this study. These findings support our hypothesis that passively expiring residual gas after laparoscopic surgery could effectively reduce PLSP.

Residual CO_2_ in the peritoneal cavity has been considered as the cause of PLSP. Previous studies concerning laparoscopic surgery under spinal anesthesia found that pneumoperitoneum gas induced shoulder pain even during surgery^24^. Our results showed that prolonged CO_2_ inflation time was associated with the higher incidence of PLSP, consistent with previous research in robotic surgery^25^. Female patients’ height may contribute to higher CO_2_ inflation volume in laparoscopic surgery^26^. This study confirmed that height was associated with PLSP after LC. Together, these results imply the association between residual pneumoperitoneum gas and PLSP. Various studies demonstrate methods for decreasing residual pneumoperitoneum CO_2_^27^, including our own previous study^28^, which used pulmonary recruitment maneuvers at the end of operation, forcing CO_2_ outflow, effectively decreasing PLSP after gynecology surgery^29^. Another study showed that abdominal wall-lift procedures for LC can decrease the incidence of PLSP, without using any inflating gas during surgery^30^. Notably, some small clinical studies showed active pneumoperitoneum gas aspiration to be effective in reducing PLSP one day post-LC^31–33^. Therefore, the role of a drain in reducing PLSP is similar to maneuvers that evacuate CO_2_ after laparoscopy.

Routine drainage is not recommended for LC. The risk of infection and more post-operative abdominal pain are proved to be associated with the use of surgical drain recently^34,35^. The primary role of drain use in laparoscopic surgery is to monitor the collection of bile or blood. There is considerable evidence that the usage of surgical drains is to allow residual gas from inflation during laparoscopy to escape^6^. In gynecologic laparoscopic procedures, randomized control trials have shown that drainage post-laparoscopy reduces the intensity and frequency of PLSP^10,27^. Similarly, a smaller randomized study also found that the incidence and severity of PLSP significantly decreased in patients with a suction drain after LC^36^. Another recent randomized study showed that patients with a drain 6, 12, 24, and 48 h post-LC had reduced shoulder pain severity^37^. Consistently, our results showed that a drain significantly reduced the incidence and pain score of PLSP in the early recovery period after LC. Most importantly, our results, along with the aforementioned studies, provide evidence that releasing CO_2_ via the drain site was strongly associated with reduced PLSP^38^, suggesting that the mechanism of PLSP is closely associated with irritation from inflating gas during laparoscopy.

This study also showed that the decision to place a surgical drain was associated with increased age and prolonged CO_2_ inflation time. Prolonged pneumoperitoneum time indicated technique difficulties. Moreover, elder patients had higher conversion rate to open cholecystectomy and more post-operative morbidity^39^. The other studies showed that elder patient was a risk factor for technique difficulty in LC^40,41^. Consequently, increased age and prolonged CO_2_ inflation time had more chance to need surgical drain to monitor the post-operative abdominal condition.

There are limitations to this study. First, this was an observational study with differences in patient characteristics between treatment and control groups. The standard for evaluating the efficacy of treatments is a prospective, randomized, and blinded trial because it ensures that both treatment and control groups are balanced for all patient characteristics. In fact, many studies cannot be randomized for ethical or practical reasons. The decision to drain or not in this study was based on surgical indication because randomization may be ethically inappropriate. Therefore, we conducted an observational study, which provided information about treatment efficacy. Dimick and colleagues have demonstrated that selection bias from unadjusted comparisons of treatment groups in observational studies leads to inaccurate results^42^. The strength of propensity score analysis is that it addresses selection bias in observational studies^43^. Hence, we attempt to replicate the design of a randomized trial by adopting a propensity score-based method. This allowed us to draw balanced groups of those who did and did not receive surgical drainage after LC. To further strengthen our methodology, we used IPTW to adjust for any known biases, which increases the power of propensity score analysis with the benefit of preserving the original sample size. This allowed us to compare the outcomes in two groups that were similar in baseline characteristics. Although propensity score analysis can balance differences between groups, it is limited to balancing unknown characteristics and confounders. To the best of our knowledge, most of the covariates relevant to PLSP in patients undergoing LC were measured in this study. Consequently, the unadjusted results were the same as the adjusted results, suggesting that the effects of PLSP-related factors were equally distributed between the groups.

Second, our results mainly showed that surgical drain use significantly reduced PLSP in the first 24 h post-LC but did not explore the association between surgical drain use and PLSP over a longer period. This was because PLSP is not a late complaint after laparoscopic procedures onset ranges from the intraoperative phase to postoperative-day seven^44^. Although the range of onset times varies, many studies have noted that the pain score of PLSP is highest in the first 24 h after surgery^27^, and most PLSP complaints occur within 24 h of surgery^44,45^. It is important to reduce the frequency of PLSP in the early post-operative phase due to the short hospitalization period for LC. This study design, that enrolled patients with PLSP within 24 h, emphasizes the early postoperative effect of drainage. Third, our results demonstrated that the incidence and severity of PLSP within post-operative 24 h were decreased in the presence of surgical drain. The reasonable assumption of this finding is due to the placement of surgical drain to reduce the residual intra-abdominal gas. Chest X-ray on post-operative day 1 for detect residual gas in each enrolled patients may be the evidence to distinguish the effects of surgical drain. Nevertheless, routine postoperative chest X-ray are not recommended in patients with uncomplicated LC due to radiation exposure.

## Conclusions

We conducted a prospective cohort study using IPTW to investigate the association between drain use and PLSP, to explain the effect of residual gas in patients undergoing LC. Our results show that surgical drain use, which relies on passive force, reduces the frequency and intensity of PLSP in the first 24 h after LC. These results suggest that strategies to evacuate residual gas completely at the end of laparoscopic procedures can improve PLSP.

## Clinical trial number

ClinicalTrials.gov database (No. NCT01095536).

## Supplementary Information

Supplementary Information

## Abbreviations

ASA: American society of anesthesiologists
CI: Confidence interval
HR: Hazard ratio
IPTW: Inverse probability of treatment weighting
LC: Laparoscopic cholecystectomy
OR: Odds ratio
PLSP: Post-laparoscopic shoulder pain
SD: Standard deviation
VRS: Verbal rating scale
