# Supplementary material for: To drain or not to drain: the association between residual intraperitoneal gas and post-laparoscopic shoulder pain for laparoscopic cholecystectomy
Source: Sci Rep. 2021 Jun 1;11:7447. doi: 10.1038/s41598-021-85714-4 (PMC8167121; doi:10.1038/s41598-021-85714-4)
Supplement: Supplementary file 1 — Supplementary Information [file 41598_2021_85714_MOESM1_ESM.docx]

**To drain or not to drain: the association between residual intraperitoneal gas and post-laparoscopic shoulder pain for laparoscopic cholecystectomy**

Shun-Chin Yang^1,#^, Kuang-Yi Chang^1,#^, Ling-Fang Wei^2^, Yi-Ming Shyr^3^, Chiu-Ming Ho^1,*^

**Supplementary Table S1 Propensity score analysis of surgical drain insertion after laparoscopic cholecystectomy**

|  | OR | 95% CI | *P* |
| --- | --- | --- | --- |
| Age | 1.05 | 1.03–1.07 | < 0.001 |
| Gender (M vs F) | 1.11 | 0.59–2.09 | 0.743 |
| Height | 1.01 | 0.98–1.05 | 0.474 |
| Weight | 1.01 | 0.98–1.03 | 0.679 |
| ASA (> 3 vs < 3) | 0.72 | 0.35–1.49 | 0.379 |
| Diabetes | 1.66 | 0.84–3.29 | 0.144 |
| Hypertension | 0.99 | 0.59–1.68 | 0.978 |
| Smoking | 2.15 | 1.11–4.16 | 0.022 |
| Fentanyl dose (> 2.5 vs < 2.5 μg kg^-1^) | 1.06 | 0.65–1.73 | 0.813 |
| Operation time (> 2 vs < 2 hours) | 1.57 | 0.94–2.62 | 0.087 |
| CO_2_ inflation time (> 1 vs < 1hour) | 4.02 | 2.36–6.87 | < 0.001 |

OR: odds ratio
